# Ragona-Scinà’s (1847) Method for, and Observations of, Simultaneous Color Contrast

**DOI:** 10.1177/2041669516643239

**Published:** 2016-04-28

**Authors:** Robert P. O’Shea, Stefano Brini, Nicholas J. Wade

**Affiliations:** School of Psychology and Exercise Science, Murdoch University, Australia; Discipline of Psychology, School of Health and Human Sciences, Southern Cross University, Coffs Harbour, Australia; School of Psychology and Exercise Science, Murdoch University, Australia; Department of Psychology and Speech-Language Pathology, University of Turku, Finland; Psychology, University of Dundee, UK

**Keywords:** Color perception, simultaneous color contrast, optical superimposition, multi-field tachistoscope, methods for research in visual perception, Ragona, history

## Abstract

In 1847, Domenico Ragona-Scinà (1820–1892) published a method of optically superimposing images using an angled piece of colored glass. He showed that if one looks at a black, filled circle through the colored glass and superimposes on it the reflection from the glass of something white, the filled circle looks tinted with the complementary color of the background: *simultaneous color contrast* or *contrast color*. Although Ragona-Scinà’s method and his observation have been cited into the 21st century, the former for its simplicity and the latter for its challenges to early theories of color vision, some errors have crept in and the phenomenon still lacks an agreed-on explanation. We provide some biographical information about Ragona-Scinà, set the method and the observation into their historical and theoretical contexts, and give a translation into English of Ragona-Scinà’s Italian-language paper.

## Introduction

Ragona-Scinà (1847b) showed that if one looks at a black, filled circle through a colored piece of glass, and then optically superimposes white light on it, by reflection from the front surface of the glass, the filled circle looks tinted with the complementary color of the glass, a phenomenon now known as simultaneous color contrast (e.g., Ekroll & Faul, 2013) or contrast color (e.g., Whittle, 2003). As we will show, Ragona-Scinà’s observation challenged early theories of color vision and his method for producing it was new for most vision researchers.

For both his observation and method, Ragona-Scinà’s paper was widely cited up to around the end of the 19th century, and more sporadically after that. Mausfeld (2003), who made the most recent citation of Ragona-Scinà we have found, observed that contrast colors “are actually still in want of a satisfactory explanation” (p. 415), a conclusion echoed by Mollon (2006) and by MacLeod (2010). To our knowledge, no translation into English exists of Ragona-Scinà (1847b), which was written in (rather difficult!) Italian. Here, we give some biographical information about Ragona-Scinà, set the paper into its historical and theoretical context, and provide a translation of Ragona-Scinà’s (1847) paper into English.

## Who Was Ragona-Scinà?

Ragona-Scinà changed his name in his published papers to Ragona by 1848. We refer to him in this paper as Ragona-Scinà.

**Figure 1.**
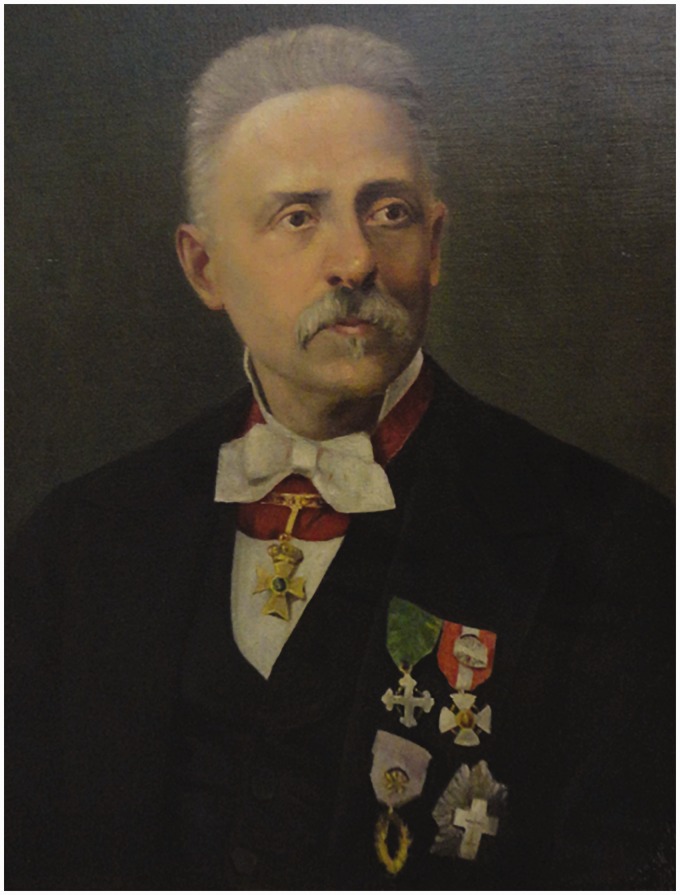
Reproduction of an oil painting of Ragona-Scinà (Chinnici, 2013).

Domenico Ragona-Scinà (Figure 1) was born in Palermo on January 20, 1820 and died on February 25, 1892 in Modena (Istituto Nazionale di Geofisica (ING), 2000). During his youth, Ragona-Scinà was close to his maternal uncle, Domenico Scinà (1765–1837; Working Group for the History of Astronomy (WGHA), 1999), an eminent physicist, mathematician, and historian (Alberti, n.d.). Scinà guided Ragona-Scinà into a scientific career, tutoring him for the last few years of his own life (WGHA).

In 1844, Ragona-Scinà received an undergraduate degree in mathematics and physics from the Royal University of Palermo (Sicily), becoming the third assistant at the Royal Observatory of Palermo (ING). In the same year, he was appointed as an adjunct professor in Physics at the Athenaeum in Palermo, elevating him to second assistant at the Observatory.

It is tempting to suppose that Ragona-Scinà added the Scinà part to his family name some time prior to 1847 to honor his uncle, but then returned to his family name, Ragona, when he felt he had discharged his educational and intellectual debt.

In 1850, Ragona-Scinà became director of the Palermo Observatory for 10 years. During his tenure, he brought the astronomical equipment of the observatory up to the cutting edge (Osservatorio astronomico di Palermo, 2015). In 1863, Ragona-Scinà became director of the Astronomical Observatory in Modena (ING), where he dedicated himself to meteorology and seismology, and similarly upgraded the extant apparatus for studying meteorology (Calanca, 2003). Ragona-Scinà was also partly responsible for founding the Italian Meteorological Society in 1876 (ING).

Although Ragona-Scinà published extensively in astronomy, meteorology, and seismology (ING), our interests are in his contributions to the methods and findings of visual perception. He wrote two papers in 1847 (Ragona-Scinà, 1847a, 1847b), one in 1859 (Ragona, 1859) and one in 1873 (Ragona, 1873), all on color vision. His method anticipated that in vision research of using angled glass for optical superimposition, which found its full flowering with multi-field tachistoscopes. These were popular in vision research from about 1910 to about the 1970s when they were largely supplanted by oscilloscopes and then computer monitors (Wade & Heller, 1997).

## What Was Ragona-Scinà’s Method and What Was His Observation?

Ragona-Scinà’s (1847b) method was to look through a colored sheet of glass tilted about 45° to a horizontal line of sight at a black, filled circle drawn on a vertical white sheet of paper while at the same time seeing the reflection, from the front surface of the glass, of a similar horizontal white sheet of paper with its own black, filled circle. For example, when Ragona-Scinà used green glass, he saw the black, filled circle on the vertical piece of paper tinted with red. The black, filled circle reflected from the horizontal piece of paper, visible near to, but not superimposed on, the filled circle seen through the glass, was tinted with green, because it is a mixture of black and green (Ragona, 1859). Ragona-Scinà (1847b) considered it remarkable that he could perceive red in the filled circle viewed through the glass when the only light his eyes were receiving from that filled circle was green and white.

Ragona-Scinà (1847a) also described another study in which he achieved the same results by using a crystal of Island spar—a bi-refringent, polarizing material—to achieve optical superimposition. Ragona-Scinà (1859) reviewed both 1847 studies, recognizing that the complementary colors he was perceiving were a phenomenon of perception, referring to them as “subjective colors” (p. 1). Moreover, he gave an explanation of the subjective colors that seems substantially acceptable today: The background of green plus white sets a general adaptation level so that an area missing green appears red (p. 3). Ragona (1873) emphasized that the reflected circle does not need to be present to experience the phenomenon and that its explanation involves processes similar to those producing complementary-color afterimages.

## What Was the Context of Ragona-Scinà’s Method?

The basic method Ragona-Scinà (1847b) used to superimpose two images had already been set forth by Giambattista della Porta in 1558 (see Porta, 1658). It was to use a piece of glass to reflect an image of an object into the eyes of someone who was also looking directly, through the piece of glass, at some other object. This technique began to be used in the middle of the 19th century in theatres in England to project ghosts—actually reflections from a large, angled, piece of glass of actors portraying ghosts in a side room to the stage—onto the main scene on the stage (Pepper’s ghost, 2015).

Interest in color contrast, particularly in Germany, was stimulated by the observations of Goethe (1810) on colored shadows. For example, Brandes (1827) used glass sheets to combine different colored patches by reflection and transmission, as did Osann (1833, 1836) and Dove (1838); Osann (1836) and Dove also used coloured glass through which patches could be observed. We have no evidence that Ragona-Scinà was aware of their work because he does not cite them. It seems common in science that academic credit does not necessarily go to the originator of an idea, either by accident (Merton, 1968; Stigler, 1980) or by design (Wike, 1973).

**Figure 2.**
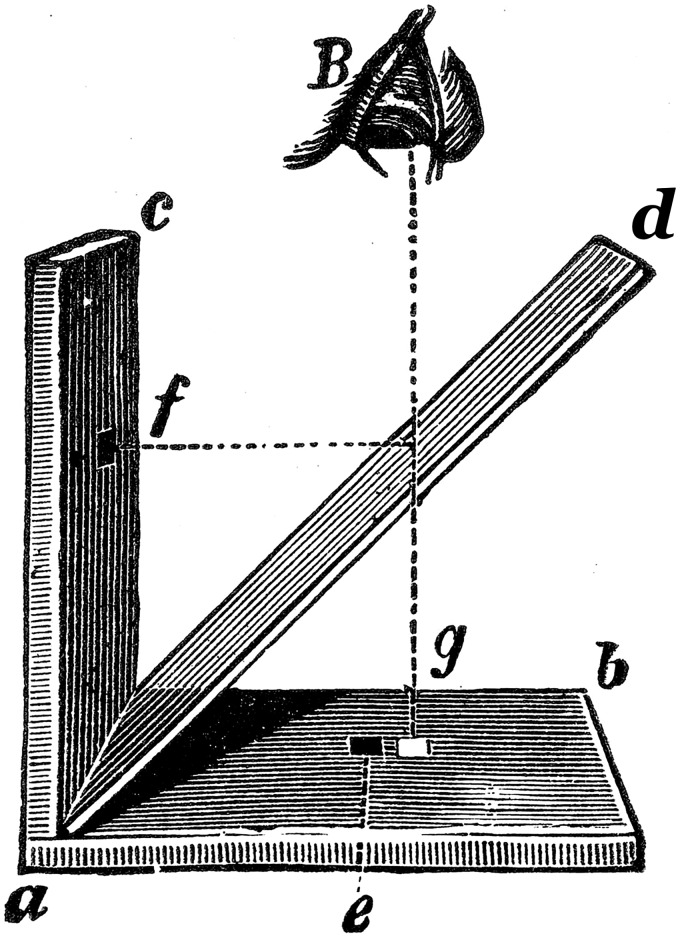
Helmholtz’s (1867, p. 405) representation of Ragona-Scinà’s method. Here is Southall’s translation of Helmholtz’s description of the figure, “*ab* and *ac* are two white paper surfaces, one horizontal, the other vertical; and *ad* is a colored plate of glass inclined to the two paper surfaces at 45°; *e* and *f* are two black spots. An observer at *B*, looking down on the apparatus from above, sees the surface *ab* through the colored glass, and the surface *ac* reflected in it. The image of *ac* coincides apparently with *ab*, and the image of the black spot *f* is at *g*, say, not far from the spot *e*.” (Helmholtz, 1924, p. 283).

Helmholtz (1867, p. 405) provided a beautiful engraving (his Figure 151, our Figure 2) depicting Ragona-Scinà’s “apparatus.” Helmholtz’s figure is incorrect in one minor aspect and rather misleading in another. The minor error is that Helmholtz showed the eye above the horizontal paper, looking down, instead of to the right, say, of the vertical paper. Of course this modification will work perfectly well if perhaps not so conveniently for an observer. Rood (1879, pp. 257–260), Tscherning (1898, p. 220), Hurst and Stocks (1916), Ostwald (1919), Allen (1924), Linksz (1952), Graham and Brown (1965, p. 462), Geldard (1972, p. 145), Hurvich (1981, p. 153), and Da Pos (1999, p. 587, Table 14) included figures similar to Helmholtz’s.

Helmholtz’s (1867) and similar figures are misleading because they imply that Ragona-Scinà (1847b) had an actual apparatus, with a piece of glass at exactly 45°, allowing very careful positioning of the black, filled circles, whereas there is no such evidence of that in Ragona-Scinà (1847b). Ragona (1859) did later construct an apparatus, but it bore no resemblance to the one depicted by Helmholtz or to the arrangement Ragona-Scinà described in 1847. The 1859 apparatus had both filled circles drawn on a single horizontal sheet with the glass held in a clamp in front of a viewing tube and relied on reflections from the front and rear surfaces of the glass, a technique used by Brandes (1827) and Dove (1838). As will be evident in our translation, it seems to us that Ragona-Scinà simply held the glass in one hand and tilted it manually.

Ragona-Scinà (1873) did finally build an apparatus similar to that illustrated by Helmholtz (1867) and used it to superimpose transmitted contrast colors in very close proximity to reflected colors, but mainly for the beauty the combined images offered viewers.

Hering (1887) also built an apparatus to provide the horizontal and vertical surfaces, and slotted in the colored glass at 45°. He replaced the black, filled circles by concentric rings of black and white. The vertical image had the opposite arrangement of blacks and whites from that of the horizontal. This does presuppose accurate construction of the apparatus and of the images, and very careful positioning of the images to ensure that the blacks of one are optically superimposed on the whites of the other and vice versa, similar to that achieved by Ragona-Scinà (1873). Sanford (1892, pp. 406–407) illustrated this sort of apparatus in a figure (p. 406). This sort of precision anticipates the optical arrangement of multi-field tachistoscopes. These were invented by Dodge (1907), who replaced the colored glass with smoked glass.

Other citations of Ragona-Scinà’s method include Oliver (1885), Axenfeld (1889) who proposed an improvement of the method using transmitted light, Breese (1899) who suggested the method could be used for optically superimposing images to produce monocular rivalry, Rivers (1900, p. 1064), Greenwood (1910, p, 204), Hayes (1911), who alluded to Hering’s development of the method for assessing color blindness, and Parsons (1915).

## What Was the Context of Ragona-Scinà’s Observation?

Good reviews of the history of other examples of simultaneous color contrast exist including by Helmholtz (1867) and by Mollon (2006). Helmholtz pointed out that Leonardo da Vinci, in 1519, was already familiar with the phenomenon in painting. Mollon attributed the first report of complementary colored shadows to von Guericke (1672); Helmholtz cited Rumford (Thompson, 1794) and Goethe (1810) for them. Mollon (2006) credited the first observation of contrast colors to Jean-Baptiste Meusnier, who reported it to Monge (1789; for a translation, see Kuehni, 1997). Helmholtz said that contrast color was carefully studied by Chevreul (1839) in fabrics, that Dove (1838) observed it with colored glass on metal mirrors, and that Meyer (1855) observed it with colored papers.

Helmholtz (1867) and later authors all accepted the validity of Ragona-Scinà’s (1847b) observation, presumably because they were able to make it themselves, as were we.

In present day visual science, we would say that the region of the retina from which one sees the color complementary to the background receives no preponderance of light consisting of wavelengths that would ordinarily appear as the complementarily color. For example, a black, filled circle seen through a green glass does not deliver any long-wavelength light to the retina where the filled circle projects. Nor does the optically superimposed white light present any preponderance of long-wavelength light. So Ragona-Scinà’s (1847b) observation was a challenge to theories of color vision such as Young’s (1804) that color is a consequence of wavelength of light.

Mollon (2006) reported that Monge (1789) developed a surprisingly modern theory of color vision, in which colors arise from the ratios of some then-unknown property of light from differently colored objects in a scene, and that the observer has to make some judgment of the properties of the illumination. This theory was designed to account for four key observations of color vision:

1. That a red object looks white when looked at through a red glass.
2. That a white object looks white when looked at through a red glass.
3. That colored shadows exist.
4. That simultaneous color contrast exists.

Again, in present day visual science, we would say Monge proposed that the brightest area in a scene appears white, even if it is contains a preponderance of a particular range of wavelength, say long wavelengths that ordinarily appear red. Other areas containing a balance of all visible wavelengths, which ordinarily appear white must shift their color in a direction complementary to white to maintain the same ratios with it. Monge’s emphasis on ratios anticipated later theories such as Land’s retinex theory (e.g., Land, 1977).

Helmholtz (1867) was unaware of Monge’s theory and developed his own. He proposed a physiological explanation residing in the retina and a higher-level psychological explanation. The physiological explanation is now known as trichromatic theory, involving ratios of activities among three receptors in the eye; it does not require any inference, conscious, or unconscious (Hurvich & Jameson, 1969). Helmholtz (1867) conceded that trichromatic theory was unable to explain Ragona-Scinà’s (1847b) observation or other examples of simultaneous color contrast (see later). The psychological explanation is known as unconscious inference, or to use Helmholtz’s words, an error, or an illusion. For example, he proposed that the green background suggests to the observer that the spot is illuminated by greenish light. Under such an illuminant, Helmholtz said, the observer makes the unconscious inference that a dark greenish spot must have arisen from a dark pink spot and that is what is perceived. This sort of thinking anticipates modern approaches to color constancy (e.g., Foster, 2011) and the effects of experience and judgment on perception (e.g., Asch, 1956). It is certainly true that judgment plays a part in making any phenomenological observation and its role is still a matter of debate (e.g., Gilchrist, 2015).

Hering (1887) reported a series of careful experiments addressing Helmholtz’s explanation. For example, he showed that the contrast colors are visible even when the observer is unaware he is looking through colored glass. Hering (1890) also had observers look at a dark spot on a white background with red glass over one eye and with blue glass over the other. Although this yielded a purple background via binocular combination of colors, when observers crossed their eyes, they saw two dark spots, one tinted with cyan and the other with orange, thereby ruling out any unconscious inference from illumination, which should have made the spots appear yellow.

Hering (1887) offered a physiological explanation of contrast colors involving reciprocal inhibition between neighboring regions of the retina. This was a notion of almost uncanny prescience, because the discovery of retinal ganglion cells having receptive fields responsive to light and dark in center and surround regions (Kuffler, 1953) and the discovery of lateral geniculate nucleus cells having receptive fields responsive to complementary colors in center and surround regions (De Valois, Smith, Kitai, & Karoly, 1958) did not emerge until the following century.

Any full explanation of the phenomenology of simultaneous color contrast will likely involve a rapprochement between bottom-up, physiological explanations and top-down, psychological explanations (MacLeod, 2010; Mollon, 2006).

## The Translation

ON CERTAIN PHENOMENA

PRODUCED BY COLORED GLASSES

LETTER OF PROF. DOMENICO RAGONA SCINÀ

TO MR. FILIPPO CIRELLI

DIRECTOR OF THE POLIGRAPHIC ESTABLISHMENT IN NAPLES

(Communicated by the author.)

Dear Mr. Cirelli.

I send a brief account of a pretty phenomenon I observed in the past few days, and of which I cannot find any report by physicists.

Draw two, equal, black, filled circles on two, white pieces of paper, then arrange the two papers at right angles, the first in a horizontal plane and the second in a vertical plane. Look at the black circle of the vertical paper through a glass of any color. When the glass is parallel to the vertical paper, the circle is seen perfectly black on a background of the same color as the glass. Slightly tilting the glass, and placing its lower part closer to the vertical card, and the upper part nearer to the eye, the circle continues to be black. But, by tilting the glass more, at a certain point, the horizontal paper reflected on the glass is projected onto the vertical paper. Then a sudden metamorphosis occurs, as the transmitted circle of the vertical paper changes its black to a very distinct color complementary to that of the glass. Simultaneously, the vertical paper, which was tinted the same color as the glass, becomes white from the superimposed reflection of the card. The reflected circle is always seen tinted the same color as the glass. That is, if the glass is tilted so that the two circles, the reflected one and the transmitted one, appear aligned next to each other on the opposite background, the reflected circle is seen as the same color as the glass, and the transmitted circle as the complementary color, on a white background. If, for example, a green glass is used, the reflected circle will be seen as green, and the transmitted circle as red; if a blue glass is used, the reflected circle will be seen as blue, and the transmitted circle as yellow, etc.

Thus, a white surface transmitted by a colored glass coinciding in the same place with a white reflected surface is seen as white; a black circle on a white background transmitted by a colored glass coinciding with a white reflected surface is seen tinted with the color complementary to that of the glass; a black circle on a white background reflected by a colored glass coinciding with a transmitted white surface is seen tinted with the same color as the glass. The experience can be produced in different ways, and it is always very enjoyable. The complementary-colored circle has a beautiful appearance when light is shone onto the transmitted paper from a conveniently located lamp. It brings wonder to note that these two circles, the reflected one and the transmitted one, which fall next to each other on the opposite backgrounds, have the same appearance, one (colored like the glass) and the other (colored like the complementary color). I have been the first to observe the following facts, part of which I have published the data, which can be verified by anyone, by moving a colored glass under a large crystal of Iceland spar through which a black circle can be seen on a white background.

In the superimposed, homologous images of the circle and glass, the circle is seen tinted with the color complementary to that of the glass, against a white background.
When superimposing opposite images, the circle is seen tinted with the same color of the glass against a white background.
When superimposing opposite images, the more intense the color of the glass, the blacker the circle appears and the less it shares the color of the glass.
The more intense the glass, the weaker is the circle tinted with the complementary color in the superimposed homologous images.
At a certain degree of intensity of the glass,^1^ in the coincidence of the homologous images, the circle becomes completely invisible, that is, at a certain degree of intensity, the colored glass acts completely as an opaque body would in similar circumstances.
This degree of intensity for the same circle is relative to the darkness of the color of the glass, and for the same glass is relative to its distance from the paper bearing the circle, and to the intensity of the latter.^2^
By moving a black piece of cardboard in the opposite direction from the colored glass, the circle tinted with the complementary color in the white background becomes black on the background of the same color of the glass; and when the intensity of the glass is such as to render the circle totally invisible in the superimposition of the homologous images, the black circle reappears completely distinct and equally black on the background colored like the glass.
By moving not a black cardboard, but another colored glass in the opposite direction, a circle tinted by the complementary color appears the same color as that of the second glass, albeit not generally, but always within the intensity relationships between the two glasses, relationships that produce in this case variable and beautiful appearances, etc., etc.

Equally it is observed in the experience we are talking about, that the more intense the glass, the weaker and duller is the transmitted circle tinted with the complementary color of the reflected paper, and the darker is the reflected colored circle with the same color of the transmitted paper; that at a certain degree of intensity of the glass the transmitted circle disappears altogether when its color is the same of the reflected paper, while the reflected circle is completely black; that moving a black cardboard onto the horizontal paper, the complementary circle becomes black on the background of the same color of the glass, or reappears when it was invisible: a very simple effect, because the black cardboard intercepts the reflected paper that coincides with the transmitted one. There is perfect correspondence in appearances arising from a colored glass, instead of a black cardboard. To give an idea of this, I include the following table. The first column gives the color of the glass that moves over the horizontal paper, the second column the tint that is assumed by this glass when seen by its reflection and projected onto the transmitted paper (vertical), the third column the tint that it is assumed by the transmitted circle when superimposed on the reflected, complementary-colored paper and on this glass.

When keeping a green glass in front of the eye

Red  …   Green  …        Black

Orange  … Yellow  …         Reddish purple

Blue  …   Green-blue  …    Dark blue.

When keeping a blue glass in front of the eye

Pale yellow … Reddish  …        Saturated yellow

Red  …    Saturated blue  …  Black

Green  …   Dark green  …      Pale green.

By moving these glasses under a large crystal of Iceland spar in the opposite direction, it is observed that there is a full match of appearances. For example, if a green glass is moving from the right to the left side, the image of the right side of the black circle will be seen as green, and the left side as red; therefore, by moving an orange glass from the left side to the right, the red image becomes purple, and the two images will be seen on a yellow background.

In the experience I am talking about, the image tinted with the complementary color, which in principle is easy to see, also becomes much weaker as the glass is tilted more, and finally disappears at some tilt, *depending on whether the glass is more or less intense*. When it has disappeared, it can be made to reappear by means of a black cardboard or of another colored glass. This disappearance is perhaps dependent upon the intensity of the reflected white surface onto which the transmitted circle is superimposed which becomes more intense with the greater obliquity of superimposition. If you look at the transmitted circle through a good bi-refringent prism, two circles tinted with the complementary color of the glass can be seen; however, tilting the glass, they do not disappear simultaneously, even when the main section of the prism is horizontal; but first one disappears, and after a sufficient interval the other image disappears too. This interval between the two disappearances perhaps depends on polarization by refraction, which the transmitted circle assumes relative to the tilt.

Trust me

Palermo 6 October 1846

*Your affectionate friend*

D. Ragona-Scinà

## Conclusion

We have provided a brief biography of Domenico Ragona-Scinà (1820–1892), described his method for making observations of simultaneous color contrast (Ragona-Scinà, 1847b), set them into their historical and theoretical contexts, and provided a translation of the paper. We hope this will prevent errors from being propagated by authors who rely on secondary sources and spark renewed interest in this unexplained phenomenon.

## Notes

1 It behooves me to warn that the most intense glass I have used shows, due to its transparency, the black circle as slightly detached from its white background.

2 V. Raccolta scientifica di Roma, anno 2, num. 19; *Raccolta fis.-chim. ital.,* etc. Vol. II. 27

## References

[bibr1-2041669516643239] Alberti, P. (n.d.). *Domenico Scinà*. Retrieved from http://www.liberliber.it/online/autori/autori-s/domenico-scina/.

[bibr2-2041669516643239] Allen F. (1924) The reflex origin of color contrast. Journal of the Optical Society of America 9: 375–388.

[bibr3-2041669516643239] Asch S. E. (1956) Studies of independence and conformity. A minority of one against a unanimous majority. *Psychological Monographs* 70: 1–70.

[bibr4-2041669516643239] Axenfeld D. (1889) Sur la vision des couleurs de contraste [On seeing contrast colors]. Archives Italiennes de Biologie 11: 81–90.

[bibr5-2041669516643239] Brandes H. W. (1827) Farbe [Color]. In: Brandes H. W. Gmelin L. Horner J. C. Muncke G. W. Pfaff C. H. (eds) Gehler's physikalisches Wörterbuch *(New ed., Vol. 4, Part 1*, Leipzig, Germany: E. B. Schwickert, pp. 39–131.

[bibr6-2041669516643239] Breese B. B. (1899) On inhibition. Psychological Monographs 3: 1–65.

[bibr7-2041669516643239] Calanca, R. (2003). *L’astronomia nel ducato estense tra il XVII ed il XIX secolo. PARTE 3 [Astronomy in the ducal period between the sixteenth and nineteenth centuries. Part 3.]*. Retrieved from http://win.eanweb.com/parte_3_trasformazione_specola.htm.

[bibr8-2041669516643239] Chevreul M.-E. (1839) De la loi du contraste simultané des couleurs et de l'assortiment des objets colorés… [The principles of harmony and contrast of colours], Paris, France: Pitois-Levrault.

[bibr9-2041669516643239] Chinnici, I. (2013). Identificato un ritratto della collezione [A portrait from the collection identified]. *INAF-Osservatorio Astronomico di Palermo, Newsletter, 17*, 1–2.

[bibr10-2041669516643239] Da Pos O. (1999) La percezione del colore [Color perception]. In: Purghé F. Stucchi N. Olivero A. (eds) La percezione visiva [Visual perception], Torino, Italy: UTET, pp. 538–591.

[bibr11-2041669516643239] De Valois R. L. Smith C. J. Kitai S. T. Karoly A. J. (1958) Response of single cells in monkey lateral geniculate nucleus to monochromatic light. Science 127: 238–239. 13495504 10.1126/science.127.3292.238

[bibr12-2041669516643239] Dodge R. (1907) An improved exposure apparatus. Psychological Bulletin 4: 10–13.

[bibr13-2041669516643239] Dove H. W. (1838) Über subjektive Komplementärfarben [On subjective complementary colors]. Annalen der Physik 121: 158–162.

[bibr14-2041669516643239] Ekroll V. Faul F. (2013) Transparency perception: The key to understanding simultaneous color contrast. Journal of the Optical Society of America A 30: 342–352. 10.1364/JOSAA.30.000342 23456110

[bibr15-2041669516643239] Foster D. H. (2011) Color constancy. Vision Research 51: 674–700. 20849875 10.1016/j.visres.2010.09.006

[bibr16-2041669516643239] Geldard F. A. (1972) The human senses, (2nd ed.) New York, NY: Wiley.

[bibr17-2041669516643239] Gilchrist A. (2015) Perception and the social psychology of ‘The Dress’. Perception 44: 229–231. 26562249 10.1068/p4403ed

[bibr18-2041669516643239] Goethe J. W. v. (1810) Zur Farbenlehre [Theory of colours], Tübingen, Germany: Cotta.

[bibr19-2041669516643239] Graham C. H. Brown J. L. (1965) Color contrast and color appearance. In: Graham C. H. (ed.) Vision and visual perception, New York, NY: John Wiley & Sons, pp. 452–478.

[bibr20-2041669516643239] Greenwood M. Jr. (1910) Physiology of the special senses, London, England: Edward Arnold.

[bibr21-2041669516643239] Hayes S. P. (1911) The color sensations of the partially color-blind: A criticism of current teaching. American Journal of Psychology 22: 369–407.

[bibr22-2041669516643239] Helmholtz H. (1867) Handbuch der physiologischen Optik [Handbook of physiological optics], Leipzig, Germany: Leopold Voss.

[bibr23-2041669516643239] Helmholtz, H. (1924). *Handbook of physiological optics. Volume II. The sensations of vision* (J. P. C. Southall, Trans.). New York, NY: Optical Society of America.

[bibr24-2041669516643239] Hering, E. (1887). Ueber die Theorie des simultanen Contrastes von Helmholtz. III. Der Spiegelcontrastversuch [On Helmholtz's theory of simultaneous contrasts. III. Mirror contrast]. *Pflügers Archiv für die gesamte Physiologie des Menschen und der Tiere, 41*, 358–367.

[bibr25-2041669516643239] Hering E. (1890) Beitrag zur Lehre vom Simultankontrast. Zeitschrift für Psychologie und Physiologie der Sinnesorgane 1: 18–28.

[bibr26-2041669516643239] Hurst G. H. Stocks H. B. (1916) Colour: A handbook on the theory of colour, (2nd. rev. ed.) London, England: Scott, Greenwood & Son.

[bibr27-2041669516643239] Hurvich L. M. (1981) Color vision, Sunderland, MA: Sinauer Associates.

[bibr28-2041669516643239] Hurvich L. M. Jameson D. (1969) Human color perception: An essay review. American Scientist 57: 143–146. 5786274

[bibr29-2041669516643239] Istituto Nazionale di Geofisica. (2000). *Ragona-Scina' Domenico*. Retrieved from http://storing.ingv.it/tromos/comments/COMM01027.htm.

[bibr30-2041669516643239] Kuehni R. G. (1997) Memoir concerning certain phenomena of vision by M. Monge “Memoire sur quelques phénomènes de la vision” Annales de Chimie 3 131–147 (1789). Color Research & Application 22: 199–203.

[bibr31-2041669516643239] Kuffler S. W. (1953) Discharge patterns and functional organization of mammalian retina. Journal of Neurophysiology 16: 37–68. 13035466 10.1152/jn.1953.16.1.37

[bibr32-2041669516643239] Land E. H. (1977) The retinex theory of color vision. Scientific American 237: 108–128. 929159 10.1038/scientificamerican1277-108

[bibr33-2041669516643239] Linksz A. (1952) Vision: Physiology of the eye, New York, NY: Grume & Stratton.

[bibr34-2041669516643239] MacLeod D. (2010) Into the neural maze. In: Cohen J. Matthen M. (eds) Color ontology and color science, Cambridge, MA: MIT Press, pp. 151–178.

[bibr35-2041669516643239] Mausfeld R. (2003) ‘Colour' as part of the format of different perceptual primitives: The dual coding of colour. In: Mausfeld R. Heyer D. (eds) Colour perception: Mind and the physical world, Oxford, England: Oxford University Press, pp. 381–430.

[bibr36-2041669516643239] Merton R. K. (1968) The Matthew effect in science. Science 159: 56–63. 5634379

[bibr37-2041669516643239] Meyer H. (1855) Über Kontrast- und Komplementärfarben [On contrast colors and complementary colors]. Annalen der Physik und Chemie 171: 170–171.

[bibr38-2041669516643239] Mollon J. (2006) Monge: The Verriest lecture, Lyon, July 2005. Visual Neuroscience 23: 297–309. 16961961 10.1017/S0952523806233479

[bibr39-2041669516643239] Monge G. (1789) Mémoire sur quelques phénomènes de la vision. Annales de Chimie 3: 131–147.

[bibr40-2041669516643239] Oliver, C. A. (1885). *A new series of Berlin wools for the scientific detection of subnormal colour-perception (color-blindness)*. Transactions of the American Ophthalmological Society. Twenty-First Annual Meeting, New London, CT, 250–260. PMC1326464 25259002

[bibr41-2041669516643239] Osann G. (1833) Beschreibung einer einfachen Vorrichtung zur Hervorbringung sogenannter complementarer Farben, und Nachweisung, dass die hiemit hervorgebrachten Farben objectiver Natur sind [Description of a simple device for producing so-called complementary colors and instructions that yield the colors objectively]. Annalen der Physik 103: 694–696.

[bibr42-2041669516643239] Osann G. (1836) Ueber Ergänzungsfarben [On supplementary colors]. Annalen der Physik 113: 287–300.

[bibr43-2041669516643239] Osservatorio astronomico di Palermo. (2015). *Wikipedia*. Retrieved from https://it.wikipedia.org/wiki/Osservatorio_astronomico_di_Palermo.

[bibr44-2041669516643239] Ostwald W. (1919) Einführung in die Farbenlehre [Introduction to the theory of color], Leipzig, Germany: Philipp Reclam.

[bibr45-2041669516643239] Parsons J. H. (1915) An introduction to the study of colour vision, Cambridge, MA: Cambridge University Press.

[bibr46-2041669516643239] Pepper's ghost. (2015). *Wikipedia*. Retrieved from https://en.wikipedia.org/wiki/Pepper%27s_ghost.

[bibr47-2041669516643239] Porta J. B. (1658) Natural magick. Book 17, of strange glasses, London, England: Thomas Young and Samuel Speed.

[bibr48-2041669516643239] Ragona D. (1859) Su taluni nuovi fenomeni di colorazione soggettiva nota [Note on certain new phenomena of subjective colour]. Atti dell Accademia di Scienze e Lettere di Palermo: Nuova Serie 3: 1–9.

[bibr49-2041669516643239] Ragona D. (1873) Su taluni fenomeni di colorazione soggettiva [Note on phenomena of subjective colour]. Memorie dell'Accademia di Scienze, Lettere ed Arti di Modena 14: 7–12.

[bibr50-2041669516643239] Ragona-Scinà D. (1847a) Nuove esperienza sulla doppia refrazione e polarizzazione della luce [New experiements on double refraction and polarization of light]. Raccolta Fisico-Chimica del Zantedeschi 2: 27–47.

[bibr51-2041669516643239] Ragona-Scinà D. (1847b) Su taluni fenomeni che presentano i cristalli colorati [On certain phenomena produced by coloured glasses]. Raccolta Fisico-Chimica del Zantedeschi 2: 207–210.

[bibr52-2041669516643239] Rivers W. H. E. (1900) Vision. In: Schafer E. A. (ed.) Text book of physiology Vol. 2, London, England: Young J. Pentland, pp. 1026–1148.

[bibr53-2041669516643239] Rood O. N. (1879) Modern chromatics, with applications to art and industry, New York, NY: D. Appleton and Company.

[bibr54-2041669516643239] Sanford E. C. (1892) A laboratory course in physiological psychology: Fourth paper: V. Vision (continued). *American Journal of Psychology* 5: 390–415. 3322051

[bibr55-2041669516643239] Stigler S. M. (1980) Stigler's law of eponymy. Transactions of the New York Academy of Sciences 39: 147–158.

[bibr56-2041669516643239] Thompson B. (1794) An account of some experiments upon coloured shadows. By Lieutenant-General Sir Benjamin Thompson, Count of Rumford, F. R. S. In a Letter to Sir Joseph Banks, Bart. P. R. S. Philosophical Transactions of the Royal Society of London 84: 107–118.

[bibr57-2041669516643239] Tscherning M. H. E. (1898) Optique physiologique *[Physiological optics]*, Paris, France: Masson and others, Booksellers of the Academy of Medicine.

[bibr101-2041669516643239] von Guericke, O. (1672). *Experimenta nova (ut vocantur) Madeburgica de vacuo spatio*. Amstelodami: John Janssonium.

[bibr58-2041669516643239] Wade N. J. Heller D. (1997) Scopes of perception: The experimental manipulation of space and time. Psychological Research 60: 227–237. 9440360 10.1007/BF00419407

[bibr59-2041669516643239] Whittle P. (2003) Contrast colours. In: Mausfeld R. Heyer D. (eds) Colour perception: Mind and the physical world, Oxford, England: Oxford University Press, pp. 115–138.

[bibr60-2041669516643239] Wike E. L. (1973) Water beds and sexual satisfaction: Wike’s law of low odd primes (WLLOP). Psychological Reports 33: 192–194.

[bibr61-2041669516643239] Working Group for the History of Astronomy. (1999). *Ragona, Domenico (1820-1892)*. Retrieved from https://web.archive.org/web/19990430025440/http://www.astropa.unipa.it/PS/RAGONA.html.

[bibr62-2041669516643239] Young T. (1804) Bakerian lecture: Experiments and calculations relative to physical optics. Philosophical Transactions of the Royal Society 94: 1–16.

